# Bioeffects of Static Magnetic Fields: Oxidative Stress, Genotoxic Effects, and Cancer Studies

**DOI:** 10.1155/2013/602987

**Published:** 2013-08-06

**Authors:** Soumaya Ghodbane, Aida Lahbib, Mohsen Sakly, Hafedh Abdelmelek

**Affiliations:** Université de Carthage, Laboratoire de Physiologie Intégrée, Faculté des Sciences de Bizerte, 7021 Jarzouna, Tunisia

## Abstract

The interaction of static magnetic fields (SMFs) with living organisms is a rapidly growing field of investigation. The magnetic fields (MFs) effect observed with radical pair recombination is one of the well-known mechanisms by which MFs interact with biological systems. Exposure to SMF can increase the activity, concentration, and life time of paramagnetic free radicals, which might cause oxidative stress, genetic mutation, and/or apoptosis. Current evidence suggests that cell proliferation can be influenced by a treatment with both SMFs and anticancer drugs. It has been recently found that SMFs can enhance the anticancer effect of chemotherapeutic drugs; this may provide a new strategy for cancer therapy. This review focuses on our own data and other data from the literature of SMFs bioeffects. Three main areas of investigation have been covered: free radical generation and oxidative stress, apoptosis and genotoxicity, and cancer. After an introduction on SMF classification and medical applications, the basic phenomena to understand the bioeffects are described. The scientific literature is summarized, integrated, and critically analyzed with the help of authoritative reviews by recognized experts; international safety guidelines are also cited.

## 1. Introduction

Living organisms are continuously exposed to the natural geomagnetic field of around 20–70 *μ*T that exists over the surface of the Earth and which is implicated in the orientation and migration of certain animal species [1].

During evolution, living organisms developed specific mechanisms for perception of natural electric and magnetic fields. These mechanisms require specific combinations of physical parameters of the applied field to be detected by biological systems. In order words, the “windows” are means by which discrete MFs are detected by biological systems. Depending on the level of structural organization these mechanisms of detection and response may be seen at different levels, for example, at membrane, cellular, or tissue levels. Sometimes the “windows” function via signal transduction cascade, brain activity, or the central nervous system [2]. The sensitivity of the biological systems to weak MF has been described elsewhere [3–5], mainly in respect to the dependence of bioeffects on the amplitude or the frequency of applied fields.

The frequency of exposure to MFs has increased with rapid advances in science and technology, such as magnetic resonance imaging (MRI) diagnosis, nuclear magnetic resonance (NMR) spectroscopy, and passenger transport systems that are based on magnetic levitation [6]. Therefore, it has become necessary to systematically elucidate the influence of MFs on the body. In an attempt to explain the biological effects of SMFs, it is useful to classify them as weak (<1 mT), moderate (1 mT to 1 T), strong (1–5 T), and ultrastrong (>5 T).

SMFs are time-independent fields whose intensity could be spatially dependent. There are four SMF parameters relevant for the interaction with a biological system: target tissue(s), magnet characteristics, magnet support device, and dosing regimen [7]. SMFs are difficult to shield and can freely penetrate biological tissues [8]. However, not only the field intensity, but also the gradient of the field has important role in biological effects of SMF [9, 10]. SMF can interact directly with moving charges (ions, proteins, etc.) and magnetic materials found in tissues through several physical mechanisms [6].

Previous research showed that SMF influences biological system in a way that causes proinflammatory changes, as well as an increase in production of reactive oxygen species (ROS) [11, 12]. Throughout the past decades, there have been several experimental results describing the effects of MFs on radical pair recombination.

As reviewed recently by Ueno and Shigemitsu [13], several biophysical and biochemical effects can be expected when biological systems are simultaneously exposed to SMFs and other forms of energy such as light and radiation [14, 15]. 

Although there is much speculation about this role, the primary mechanism is thought to be the result of oxidative stress, that is, free radical generation via Fenton reaction, which is the iron-catalyzed oxidation of hydrogen peroxide (H_2_O_2_) [16–18].

Recent advance of biological science and technology can help us understand MF effects more clearly. Studies on the biological effects of MFs have resulted in significant developments in the medical applications of SMF as well as EMF, after the development of high-strength superconducting magnets. The mainstays of such medical applications are transcranial magnetic stimulation (TMS) and MRI. These techniques have also contributed much to the amazing progress made in understanding brain functions. A guideline for exposure of the human body to SMFs set by the international commission on nonionizing radiation protection (ICNIRP) [19] suggests 2 T as the ceiling value for body parts, except for arms and legs, in occupational exposure. In the application of clinical MRI, the current exposure level is confirmed to be less than or equal to 2 T. In SMFs at this strength it is not feasible to obtain resonance images, except for hydrogen atoms. There are several reports that strong SMF effects play significant roles in endogenous and exogenous ROS generations. Based on advanced studies of SMF effects on oxidative stress reactions, the potentially hazardous effect of SMF on living organisms is that exposure to SMF can increase the activity, concentration, and life time of paramagnetic free radicals, which might cause oxidative stress, genetic mutation, and/or apoptosis [20–23]. In particular, SMF exposure initiates an iron-mediated process that increases free radical formation in brain cells, leading to the breaking of DNA strands and cell death.

Genotoxic effects of exposure to static magnetic fields have been mostly examined in cell cultures [24]. Few *in vivo* studies of genotoxicity or possible effects on other carcinogenic processes have been carried out. Animal studies are often used in the evaluation of suspected human carcinogens [25] either screening for an increased incidence of spontaneous tumors or of the incidence of tumors induced by known carcinogens.

The earlier literature has been summarized by WHO [26], Kowalczuk et al. [27] and ICNIRP [28, 29], Repacholi and Greenebaum [30], IARC [31], ICNIRP [32], McKinlay et al. [33], and Dini and Abbro [34] whilst more recent studies have been reviewed by Okano [22], Phillips et al. [35], and Ueno and Okano [36].

The focus of this review is on recent studies, where possible. These studies are covered under three main sections: free radical generation and oxidative stress, apoptosis and genotoxicity, and cancer.

The objective of this review is to describe and shed light on some of the most recent information on the biological effects and medical applications of magnetic fields. A discussion of possible implications of these effects on biological systems is also provided.

## 2. Oxidative Stress

Biological free radicals are most commonly oxygen or nitrogen based with an unpaired electron, leading to the terms ROS, such as superoxide anion (O^2−^), hydroxyl radical (OH^•^) and singlet oxygen (_1_O^2^), or “reactive nitrogen species (RNS)”, such as nitric oxide (NO) [37]. The ROS and RNS play significant roles in immunological defense [38], intracellular signaling [39], and intercellular communication [40]. It is assumed that SMF could change the lifetime of radical pairs, yields of cage products, and escape products. If an SMF affects cells through the radical pair mechanism, an SMF influences the spin of electrons in free radicals, which may lead to changes in chemical reaction kinetics and possibly altering cellular function [41].

### 2.1. Moderate-Intensity Static Magnetic Fields and Oxidative Stress

There are several reports showing that moderate SMF could influence the ROS modulation (generation/reduction) from enzymatic reactions in cell-free solutions. The SMF effects also play significant roles in the endogenous and exogenous ROS modulation in biological systems, *in vitro* and *in vivo*.

Amara et al. [42] investigated the effect of SMF exposure on testicular function and antioxidant status in rats. Exposure to SMF (128 mT; 1 h/day for 30 days) has no effect on epididymal sperm count, spermatozoa motility, and genital organ weight. In contrast, SMF induces an increase of malondialdehyde (MDA) in the testis. In the gonad, SMF decreases the catalase (CAT), glutathione peroxidase (GPx), and mitochondrial Mn-superoxide dismutase (Mn-SOD) activities. However, cytosolic CuZn-SOD activity is unaffected.

The latter group also investigated the effects of SMF (128 mT, 1 h/day during 30 consecutive days) exposure on the antioxidative enzymes activity and MDA concentration in male rat brain [43]. The exposure of rats to SMF decreased the GPx, CuZn-SOD, and CAT activities in frontal cortex. The same treatment decreased the CuZn-SOD and Mn-SOD activities in hippocampus. However, the glutathione levels remained unchanged in both brain structures. In the hippocampus, SMF-exposure increased MDA concentration. These results indicated that exposure to SMF induced oxidative stress in rat hippocampus and frontal cortex.

SMF exposure alters antioxidant enzyme activity and the labile zinc fraction in THP1 cells (monocyte line) [44]. Cell culture flasks were exposed to SMF (250 mT) during 1 h, 2 h, and 3 h. Cell viability was slightly lower in SMF-exposed groups compared to a sham-exposed group. However, SMF exposure failed to alter MDA, GPx, CAT, and SOD levels even by 3 h of exposition. Cells stained with zinc-specific fluorescent probes zinpyr-1 showed a decrease of labile zinc fraction in all groups exposed to SMF. SMF exposure (250 mT, during 3 h) did not cause oxidative stress in THP1 cells but altered the intracellular labile zinc fraction.

Chater et al. [45] evaluated the effects of exposure to SMF on some parameters indicative of oxidative stress in pregnant rat. Exposure to SMF (128 mT; 1 h/day from day 6 to day 19 of pregnancy) failed to alter plasma MDA and GPx activity. Moreover the same treatment did not alter liver concentration of MDA and kidney activities of GPx CAT and SOD. By contrast, SMF induced an increase of liver GSH content. Similar results were reported by Ghodbane et al. [46] who show that liver GSH concentrations were significantly higher in SMF exposed rats than in the controls, indicating an adaptive mechanism to electromagnetic pollution. GSH levels can be increased due to an adaptive mechanism to slight oxidative stress through an increase in its synthesis. However, a severe oxidative stress may decrease GSH levels due to the loss of adaptive mechanisms and the oxidation of GSH to GSSG.

Exposure to SMF (128 mT; 1 h/day for 5 days) induces a decrease of selenium levels in kidney, muscle, and brain with a decrease of GPx activities in kidney and muscle. By contrast, SMF exposure increased total GSH levels and total SOD activities in liver, while glutathione reductase (GR) activity is unaffected. Selenium supplementation (Na_2_SeO_3_, 0.2 mg/L, in drinking water for 4 weeks) in SMF-exposed rats restored selenium levels in kidney, muscle, and brain and elevated the activities of GPx in kidney and muscle to those of control group. In the liver, selenium supplementation failed to bring down the elevated levels of total GSH and SOD activities [46]. Thus, subacute exposure to SMF altered the antioxidant response by decreasing tissues selenium contents, while selenium supplementation ameliorates antioxidant capacity in rat exposed to SMF. Regarding the fate of selenium administration in SMF-exposed rats, it may be assumed that this element minimizes the oxidative stress induced by SMF.

Previous data implicated the SMF in free radical production, like superoxide anions in different cells and organs [22, 47, 48]. However, Ghodbane et al. [49] showed that SMF exposure failed to alter plasma TBARs and total thiol groups, indicating an adaptive mechanism to slight oxidative stress caused by electromagnetic field as previously shown by Chater et al. [45]. By contrast, Amara et al. [50] showed an increase in MDA level in liver and kidney, indicating oxidative stress under SMF (128 mT, 1 h/day during 30 consecutive days). This discrepancy may be explained by the intensity and the duration of the exposure. The cellular and molecular modifications induced when SMFs interact with biological materials are, however, dependent on the duration of exposure, intensity, tissue penetration, and the type of cells [51].

Moreover, Ghodbane et al. [49] evaluated the effect of selenium (Se) supplementation in SMF-exposed rats. Pretreatment with Se (Na_2_SeO_3_, 0.2 mg/L, for 30 consecutive days, *per os*) prevented plasma *α*-tocopherol and retinol decrease induced by SMF exposure.

Amara et al. [50] examined the effect of zinc supplementation on the antioxidant enzymatic system, lipid peroxidation and DNA oxidation in SMF-exposed rats. The exposure of rats to SMF (128 mT, 1 h/day during 30 consecutive days) decreased the activities of GPx, CAT, and SOD activities and increased MDA concentration in liver and kidneys. Zinc supplementation (ZnCl_2_, 40 mg/L, *per os*) in SMF-exposed rats restored the activities of GPx, CAT, and SOD in liver to those of control group. However, only CAT activity was restored in kidney. Moreover, zinc administration was able to bring down the elevated levels of MDA in the liver but not in kidneys. The authors suggested that zinc supplementation minimizes oxidative damage induced by SMF in rat tissues.

The mechanism by which SMF induced oxidative stress in rat tissues is not well understood. A change in radical pair recombination rates is one of the few mechanisms by which an SMF can interact with biological systems such as a cell-free system. The SMF increases the concentration and/or lifetime of free radicals that escape from the radical pair so that the critical radical concentration, needed to initiate membrane damage and cause cell lysis, is reached sooner [22].

Exposure to SMF (128 mT, 1 h/day, during 5 consecutive days) induced sympathetic neurons system hyperactivity associated with hypoxia-like status [52] and elevated plasma corticosterone and metallothionein concentrations and enhanced apoptosis [53, 54]. Hashish et al. [8] indicate that there is a relation between the exposure to SMF and the oxidative stress through distressing redox balance leading to physiological disturbances. SMF exposure induced probably the disruption of mineral divalent element homeostasis, contributing to their deficiency in tissues [43, 44, 46, 50]. Agay et al. [55] have demonstrated that alteration of antioxidant trace elements (Zn, Se, and Cu) disrupts the activities of antioxidant enzymes. Duda et al. [56] reported a change in liver and kidneys concentration of copper, manganese, cobalt and iron in rats exposed to static and low-frequency magnetic fields. SMF probably induces a conformational change of antioxidant enzymes that leads to loss of their catalytic activity [56].

A few studies concerning the supplemental antioxidants vitamins C and E have focused on the preventive and curative properties in damage induced by SMF exposure [57]. Jajte et al. [58] reported the effect of melatonin and vitamin E on the level of lipid peroxidation in rat blood lymphocytes exposed to iron ions and/or SMF. When cells were treated with melatonin or vitamin E and then exposed to iron ions and SMF, the level of lipid peroxidation was significantly reduced.

Sullivan et al. [59] reported that SMF (230–250 mT) exposure stimulates ROS production in human fetal lung cells (WI-38) during the first 18 h period when cells are attaching to the culture vessel. These results support the hypothesis that increased ROS formation may account for SMF effects on cell attachment. However, SMF decreases growth in cell when the increase in ROS was abated, suggesting that other mechanisms account for SMF effects on cell growth.

Kabuto et al. [60] showed that an SMF (5–300 mT for 40 min) had no effect on the accumulation of TBARS in mouse brain homogenates induced by FeCl_3_. In contrast, when the homogenates were incubated with FeCl_3_ in an SMF (2–4 mT), the accumulation of TBARS was decreased. The accumulation of TBARS in phosphatidylcholine solution incubated with FeCl_3_ and H_2_O_2_ was also inhibited by the SMF exposure. These results suggest that the SMF could have an inhibitory effect on Fe^2+^-induced lipid peroxidation, and the effectiveness of this SMF suppression on Fe^2+^-induced ROS generation is restricted to a “window” of field intensity of 2 to 4 mT.

Currently, environmental and industrial pollution causes multiple stress conditions; the combined exposure to magnetic field and other toxic agents is recognized as an important research area, with a view to better protecting human health against their probable unfavorable effects. Amara et al. [61] investigated the effect of coexposure to SMF and cadmium (Cd) on the antioxidant enzymes activity and MDA concentration in rat skeletal and cardiac muscles. The exposure of rats to SMF (128 mT, 1 h/day during 30 consecutive days) decreased the activities of GPx and CuZn-SOD in heart muscle. Exposure to SMF increased the MDA concentration in rat cardiac muscle. The combined effect of SMF and Cd (CdCl_2_, 40 mg/L, *per os*) disrupted more the antioxidant enzymes activity in rat skeletal and cardiac muscles.

The combined effect of SMF (128 mT, 1 hour/day for 30 consecutive days) and CdCl_2_ (40 mg/L, *per os*) decreased SOD activity and glutathione level and increased MDA concentration in frontal cortex as compared with Cd-exposed rats [62].

In pregnant rats coexposed to cadmium (CdCl_2_, 3.0 mg/Kg body weight) and SMF (128 mT/1 h/day) for 13 consecutive days as from the 6th to 19th day of gestation, no effects on activities of antioxidant were observed in both tissues compared to cadmium-treated group [63]. However, the association between SMF and Cd decreased plasma MDA concentration, suggesting that a homeostatic defense mechanism was activated when SMF was associated to Cd in pregnant rats.

### 2.2. Strong and Ultrastrong Static Magnetic Fields and Oxidative Stress

Although strong SMF is supposed to have the potential to affect biological systems, the effects have not been evaluated sufficiently.

Sirmatel et al. [64] investigated the effects of a high-strength magnetic field produced by an MRI apparatus on oxidative stress. The effects of SMF (1.50 T) on the total antioxidant capacity (TAC), total oxidant status (TOS), and oxidative stress index (OSI) in male subjects were investigated. In this study, 33 male volunteers were exposed to SMF for a short time, and the TAC, TOS, and OSI of each subject were determined using the methods of Erel. Magnetic field exposure was provided using a magnetic resonance apparatus; radiofrequency was not applied. TAC showed a significant increase in postexposures compared to preexposures to the magnetic field (*P* < 0.05). OSI and TOS showed a significant decrease in postexposures compared to preexposures to SMF (for each of two, *P* < 0.01). The 1.50 T SMF used in the MRI apparatus did not yield a negative effect; on the contrary, it produced the positive effect of decreasing oxidative stress in men following short-term exposure.

The Nakagawa research group [65, 66] measured and evaluated a ROS scavenger, metallothionein (MT), a ROS product, and lipid peroxidation in the liver, kidneys, heart, lung, and brain of 8-week-old male BALB/c mice *in vivo*. The mice were exposed to an SMF of 3.0 and 4.70 T for 1–48 h. A 4.70 T SMF exposure for 6–48 h increased both MT and lipid peroxidation levels in the liver alone. A 3.0 T SMF exposure for 1–48 h did not induce any changes in both MT and lipid peroxidation levels in all the tissues. A single subcutaneous injection of CCl_4_ (0.5 mL/kg) increased both MT and lipid peroxidation levels in the liver, and the combination of CCl_4_ administration and a 4.70 T SMF for 24 h potentiated both MT and lipid peroxidation levels. The increase in activities of both glutamic-oxaloacetic transaminase (GOT) and glutamic-pyruvic transaminase (GPT) caused by CCl_4_ administration was also enhanced by the SMF exposure. It is concluded that exposure to a high SMF induces the increase of both MT and lipid peroxidation levels in the liver of mice and enhances the hepatotoxicity caused by CCl_4_ injection.

## 3. Genotoxicity, DNA Damage, and Apoptosis

Health and environmental concerns have been raised because the SMF effects on oxidative stress leading to genetic mutation and apoptosis/necrosis have been found. It seems to take place from free radical generation.

Several experiments have been shown, and they discussed how SMF can influence the immune function or oxidative DNA damage via the ROS formation process.

One possibility is that DNA is damaged by free radicals that are formed inside cells. Free radicals affect cells by damaging macromolecules, such as DNA, protein, and membrane lipids. Several reports have indicated that SMF enhances free radical activity in cells [67–71], particularly via the Fenton reaction [70]. The Fenton reaction is a process catalyzed by iron in which hydrogen peroxide, a product of oxidative respiration in the mitochondria, is converted into hydroxyl free radicals, which are very potent and cytotoxic molecules.

### 3.1. Genotoxic Effects of Moderate-Intensity Static Magnetic Fields

Amara et al. [44] investigated the effect of SMF exposure in DNA damage in THP1 cells (monocyte line). Cell culture flasks were exposed to SMF (250 mT) during 1 h, 2 h, and 3 h. The results showed that cell viability was slightly lower in SMF-exposed groups compared to a sham-exposed group. DNA analysis by single cell gel electrophoresis (comet assay) revealed that SMF exposure did not exert any DNA damage by 1 and 2 h. However, it induced a low level of DNA single strand breaks in cells after 3 h of exposition. To further explore the oxidative DNA damage, cellular DNA was isolated, hydrolyzed, and analyzed by HPLC-EC. The level of 8-oxo-7,8-dihydro-2′-deoxyguanosine (8-oxodGuo) remained unchanged compared to the sham-exposed group (+6.5%, *P* > 0.05). The results showed that SMF exposure (250 mT, during 3 h) did not cause oxidative stress and DNA damage in THP1 cells.

Exposure of rats to SMF (128 mT, 1 h/day during 30 consecutive days) increased metallothioneins level in frontal cortex, while the 8-oxodGuo concentration remained unaffected, indicating the absence of DNA oxidation. Metallothionein induction protected probably DNA against oxidative damage [43]. The same treatment elevated the 8-oxodGuo in kidneys but not in liver. Zinc supplementation (ZnCl_2_, 40 mg/L, *per os*) attenuated DNA oxidation induced by SMF in kidneys to the control level [50].

Simultaneous exposure of rat lymphocytes to a 7 mT SMF and ferrous chloride (FeCl_2_) caused an increase in the number of cells with DNA damage [72, 73]. No significant differences were observed between unexposed lymphocytes and lymphocytes exposed to a 7 mT SMF or FeCl_2_ (10 mg/mL). However, when lymphocytes were exposed to a 7 mT static magnetic field and simultaneously treated with FeCl_2_, there was a significant increase in the percentage of apoptotic and necrotic cells accompanied by significant alterations in cell viability.

However, an increasing number of evidence indicates that SMFs are capable of altering apoptosis, mainly through modulation of Ca^2+^ influx. Tenuzzo et al. [74] observed that exposure to a 6-mT SMF affects the apoptotic rate in isolated human lymphocytes, the expression and distribution of pro- and antiapoptotic genes, and the concentration of intracellular Ca^2+^. They also suggest that modulation of the apoptotic rate is not a consequence of the direct physical interaction between the field and the apoptotic inducers but the final result of multiple perturbations of Ca^2+^-regulated activity and gene-related transcription factors and membrane components, which collectively affect the apoptotic response.

SMFs above 600 mT were found to decrease the extent of cell death by apoptosis induced by several agents in different human cell systems of U937 and CEM cells in an intensity-dependent fashion, reaching a plateau at 6 mT [75]. The protective effect was found to be mediated by the ability of the fields to enhance Ca^2+^ influx from the extracellular medium; accordingly, it was limited to those cell systems where Ca^2+^ influx was shown to have an antiapoptotic effect. In addition to the SMF-enhancing effect on [Ca^2+^]i, as a mechanism of the rescue of damaged cells, it was recently proposed that SMF-produced redox alterations may be part of the signaling pathway leading to apoptosis antagonism [76].

Flipo et al. [77] examined the *in vitro* effects of SMFs on the cellular immune parameters of the C57BI/6 murine macrophages, spleen lymphocytes, and thymic cells. The cells were exposed *in vitro* for 24 h at 37°C, 5% CO_2_, to 25–150 mT SMF. Exposure to the SMF resulted in the decreased phagocytic uptake of fluorescent latex microspheres, which was accompanied by an increased intracellular Ca^2+^ level in macrophages. Exposure to SMF decreased mitogenic responses in lymphocytes, as determined by incorporation of [^3^H] thymidine into the cells. This was associated with the increased Ca^2+^ influx in concanavalin A-stimulated lymphocytes. Furthermore, exposure to SMF produced markedly increased apoptosis of thymic cells, as determined by flow cytometry. Overall, *in vitro* exposure of immunocompetent cells to 25–150 mT SMF altered several functional parameters of C57BI/6 murine macrophages, thymocytes, and spleen lymphocytes [77].

Apoptosis induced by magnetic field in female rats was investigated by using the Tdt mediated dUTP nick-end labeling (TUNEL) assay in thymus, liver, and kidneys [54]. Following subacute exposure to SMF, morphological examinations revealed numerous apoptotic cells in thymus characterized by nuclear chromatin condensation and fragmentation. The density of the apoptotic cells was significant in cortical zone, than in the medullar zone. By contrast, no labeling was found in liver and kidneys following SMF-exposure. Thus, it may be concluded that SMF induced apoptosis in thymic cell death but not in the liver and kidneys. Although the mechanisms by which SMF initiates apoptosis in thymocytes are presently not known, and reactive oxygen species are likely to play a role.

Ishisaka et al. [78] investigated the effects of an SMF (25 mT for 1 h) on the apoptosis of human leukemic cell line (HL-60) induced by exogenous H_2_O_2_. The H_2_O_2_ induced a rapid DNA fragmentation and a slow decrease in viability of HL-60 cells. However, the SMF itself (6 mT for 18 h) did not exert any effect on the H_2_O_2_-induced DNA fragmentation or viability.

HL-60 cells were exposed to SMF of 6 mT with or without DNA topoisomerase I inhibitor, camptothecin for 5 h. SMF alone did not produce any apoptogenic or neurogenic effect in HL-60 cells [79]. SMFs alone or in combination with camptothecin did not affect overall cell viability, but they accelerated the rate of cell transition from apoptosis to secondary necrosis after induction of apoptosis by camptothecin.

In addition, Teodori et al. [80, 81] reported that a uniform SMF (6 mT for 18 h; 8 and 30 mT for 3 h) did not affect viability of human glioblastoma cells. However, a uniform SMF of 300 mT for 3 h increased apoptosis. The interference of the SMF (6 mT for 18 h) with physical (heat shock) or chemical (etoposide, VP16) induced apoptosis may be related to oxidative stress.

Potenza et al. [82] described the effects of SMF on cell growth and DNA integrity of human umbilical vein endothelial cells (HUVECs). The authors investigated that a 4 h exposure of HUVECs to SMFs of moderate intensity (300 mT) induced a transient DNA damage both at the nuclear and mitochondrial levels. This response was paralleled by increased mitochondrial DNA content and mitochondrial activity and by a higher expression of some genes related to mitochondrial biogenesis 24 h after SMF exposure.

Hao et al. [83] investigated whether SMFs (8.8 mT, for 12 h) can enhance the killing effect of adriamycin (ADM) in human leukemia cells (K562). The authors showed that SMF exposure enhanced the cytotoxicity potency of ADM on K562 cells and suggested that the decrease in P-glycoprotein expression may be one reason underlying this effect.

Sarvestani et al. [84] evaluated the influence of an SMF (15 mT, for 5 h) on the progression of cell cycle in rat BMSCs. The cells were divided into two groups. One group was exposed to SMF alone, whereas the other group was exposed to X-rays before SMF exposure. The population of cells did not show any significant difference in the first group, but the second group exposed to acute radiation before SMF exposure showed a significant increase in the number of cells in the G2/M phase. The SMF intensified the effects of X-ray exposure, whereas SMF alone did not have any detectable influence on cell cycle.

### 3.2. Genotoxic Effects of Strong and Ultrastrong Static Magnetic Fields

It is generally accepted that static fields below 1 T are not genotoxic [32, 33, 85]. However, a recent study by Suzuki et al. [86] reported a significant, time- and dose-dependent increase in micronucleus frequency in mice exposed to static magnetic fields of 2, 3, or 4.7 T for 24, 48, or 72 h, using a standard micronucleus assay. Bone marrow smears were taken immediately after exposure, and the frequency of micronucleated polychromatic (immature) erythrocytes was scored. Micronucleus frequency was significantly increased following exposure to 4.7 T for all three time periods and to 3 T after exposure for 48 or 72 h, whereas exposure to 2 T had no significant effect. The authors suggest that exposure to higher fields may have induced a stress reaction or directly affected chromosome structure or separation during cell division.

The clinical and preclinical uses of high-field intensity (HF, 3 T and above) magnetic resonance imaging (MRI) scanners have significantly increased in the past few years. However, potential health risks are implied in the MRI and especially HF MRI environment due to high-static magnetic fields, fast gradient magnetic fields, and strong radiofrequency electromagnetic fields. The genotoxic potential of 3 T clinical MRI scans in cultured human lymphocytes *in vitro* was investigated by analyzing chromosome aberrations (CA), micronuclei (MN), and single-cell gel electrophoresis [87]. Human lymphocytes were exposed to electromagnetic fields generated during MRI scanning (clinical routine brain examination protocols: three-channel head coil) for 22, 45, 67, and 89 min. A significant increase in the frequency of single-strand DNA breaks following exposure to a 3 T MRI was observed. In addition, the frequency of both CAs and MN in exposed cells increased in a time-dependent manner. The frequencies of MN in lymphocytes exposed to complex electromagnetic fields for 0, 22, 45, 67, and 89 min were 9.67, 11.67, 14.67, 18.00, and 20.33 per 1000 cells, respectively. Similarly, the frequencies of CAs in lymphocytes exposed for 0, 45, 67, and 89 min were 1.33, 2.33, 3.67, and 4.67 per 200 cells, respectively. These results suggest that exposure to 3 T MRI induces genotoxic effects in human lymphocytes.

Schreiber et al. [88] reported no mutagenic and comutagenic effects of magnetic fields used for MRI.

Schwenzer et al. [89] evaluated the effects of the static magnetic field and typical imaging sequences of a high-field magnetic resonance scanner (3 T) on the induction of double strand breaks (DSBs) in two different human cell lines. Human promyelocytic leukemia cells (HL-60) and human acute myeloid leukemia cells (KG-1a) were exposed to the SMF alone and to turbo spin-echo (TSE) and gradient-echo (GE) sequences. Flow cytometry was used to quantify gammaH2AX expression of antibody-stained cells as a marker for deoxyribonucleic acid DSBs one hour and 24 hours after magnetic field exposure. X-ray-treated cells were used as positive control. Neither exposure to the SMF alone nor to the applied imaging sequences showed significant differences in gammaH2AX expression between exposed and sham-exposed cells. X-ray-treated cells as positive control showed a significant increase in gammaH2AX expression. SMF alone and MRI sequences at 3 T have no effect on the induction of DSBs in HL-60 and KG-1a cells.

The effects of SMF (4.70 T) on the frequency of micronucleated cells in CHL/IU cells induced by mitomycin C (MMC) were studied *in vitro* [90]. The cells were simultaneously exposed to SMF and MMC for 6 h, and then the cells were cultured in normal condition for the micronucleus expression up to 66 h. Exposure to SMF for 6 h significantly decreased the frequency of MMC-induced micronucleated cell expression after culture periods of 18, 42, 54, and 66 h. These results suggested that SMF (4.70 T) might have exerted an influence on the DNA damage stage produced by MMC rather than on the formation of micronuclei during the stage following MMC-induced DNA damage.

Kimura et al. [91] examined the effect of 3 or 5 T SMF on gene expression in the experimental model metazoan *Caenorhabditis elegans*. In addition, transient induction of *hps12* family genes was observed after SMF exposure. The small-*hps* gene, *hps16*, was also induced but to a much lesser extent, and the lacZ-stained population of *hps16-1::lacZ* transgenic worms did not significantly increase after SMF exposure with or without a second stressor, mild heat shock. Several genes encoding apoptotic cell death activators and secreted surface proteins were upregulated after ionizing radiation (IR), but they were not induced by SMF. The RT-PCR analyses for 12 of these genes confirmed the expression differences between worms exposed to SMF and those exposed to IR. In contrast to IR, exposure to high SMFs did not induce DNA double-strand breaks or germ line cell apoptosis during meiosis. These results suggest that the response of *C. elegans* to high SMFs is unique and capable of adjustment during long exposure and that this treatment may be less hazardous than other invasive treatments and drugs.

Koana et al. [92] examined the genotoxic effects of a 5 T SMF for 24 h in a DNA-repair defective mutant of *D. melanogaster* using the somatic mutation and recombination test (SMART) [93] because this test was useful to detect the mutagenic activity of SMF and EMF. They reported that the SMF exposure increased the frequency of mutation in *mei-41* heterozygotes and that the increase was suppressed to control levels by supplementation with vitamin E, which is a lipid-soluble antioxidant and a nonspecific radical scavenger.

An *Escherichia coli* (*E. coli*) mutation assay was used to assess the mutagenic effects of strong static magnetic fields [21]. Various mutant strains of *E. coli* were exposed up to 9 T for 24 h, and the frequencies of rifampicin-resistant mutations were then determined. The expression of the soxS::lacZ fusion gene was assessed by measuring b-galactosidase activity. The results for survival or mutation obtained with the wild-type *E. coli* strain GC4468 and its derivatives defective in DNA repair enzymes or redox-regulating enzymes showed no effect of exposure. On the other hand, the mutation frequency was significantly increased by exposure to SMF of 9 T in soxR and sodAsodB mutants, which are defective in defense mechanisms against oxidative stress.

Ikehata et al. [94] examined possible mutagenic and comutagenic effects of strong static magnetic fields using the bacterial mutagenicity test. No mutagenic effect of SMFs up to 5 T was detected using four strains of *Salmonella typhimurium* and *E. coli* WP2 uvrA. The mutation rate in the exposed group was significantly higher than in the nonexposed group when cells were treated with N-ethyl-N0-nitro-N-nitrosoguanidine, N-methyl-N0-nitro-N-nitrosoguanidine, ethylmethanesulfonate, 4-nitroquinoline-N-oxide, 2-amino-3-methyl-3H-imidazo[4,5-f]quinolone, or 2-(2-furyl)- 3-(5-nitro-2-furyl) acrylamide.

Long-term exposure to a 10 T SMF for up to 4 days did not affect cell growth rate or cell cycle distribution in Chinese hamster ovary CHO-K1 cells [95]. Exposure to SMF alone did not affect micronucleus formation. In X-ray irradiated cells, exposure to a 1 T SMF also did not affect micronucleus formation, but exposure to a 10 T SMF resulted in a significant increase in micronucleus formation induced after a 4 Gy exposure. One of the mechanisms of this effect is attributable to the 10 T SMF-induced oxidative DNA damage.

## 4. Cancer Studies

Many researchers have observed the effects of SMFs on tumor cells, particularly the inhibiting effects. They have used SMF as an entry point for investigating biological effects. In order to reduce the toxicity and resistance of single anticancer drugs, a variety of unified treatments were required. The synergy of magnetic fields and anticancer drugs was one of the methods that provides a new strategy for the effective treatment of cancer.

### 4.1. Moderate-Intensity Static Magnetic Fields and Cancer

Gray et al. [71] evaluated the effects of non-uniform 110 mT SMF for four 4 h periods, with 8–12 h between each exposure, and doxorubicin (10 mg/kg, i.p.) on female B6C3F1 mice with transplanted mammary adenocarcinoma. Their results revealed that the groups exposed to SMF combined with doxorubicin achieved significantly greater tumor regression than the group treated with adriamycin (ADM) alone. In an *in vivo* experiment, mice bearing murine Lewis lung carcinomas (LLCs) were treated with 3 mT SMF for 35 min/day and cisplatin (3 mg/kg, i.p.) [96]. The survival time of mice treated with cisplatin and SMF was significantly longer than that of mice treated only with cisplatin or SMF exposure. These results show that SMF can inhibit the proliferation of cancer cells, and the killing effects of SMF combined with antineoplastic drugs on cancer cells are greater than those of SMFs or anticancer drugs alone. These observations suggest a potential strategy for chemotherapy, that is, the combination therapy of SMFs and chemotherapeutic drugs. However, so far it remains unclear which mechanism underlies the killing effects of SMFs combined with chemotherapy drugs on cancer cells.

Sun et al. [97] evaluated the ability of 8.8 mT SMFs to enhance the *in vitro* action of a chemotherapeutic agent, paclitaxel, against K562 human leukemia cells. The authors analyzed the cell proliferation, cell cycle distribution, DNA damage, and alteration of cell surface and cell organelle ultrastructure after K562 cells were exposed to paclitaxel in the presence or absence of SMF. the results showed that, in the presence of SMF, the efficient concentration of paclitaxel on K562 cells was decreased from 50 to 10 ng/mL. Cell cycle analysis indicated that K562 cells treated with SMF plus paclitaxel were arrested at the G2 phase, which was mainly induced by paclitaxel. Through comet assay, the authors found that the cell cycle arrest effect of paclitaxel with or without SMF on K562 cells was correlated with DNA damage. The results of atomic force microscopy and transmission electron microscopy observation showed that the cell ultrastructure was altered in the group treated with the combination of SMF and paclitaxel, holes and protuberances were observed, and vacuoles in cytoplasm were augmented. The authors indicated that the potency of the combination of SMF and paclitaxel was greater than that of SMF or paclitaxel alone on K562 cells, and these effects were correlated with DNA damage induced by SMF and paclitaxel. Therefore, the alteration of cell membrane permeability may be one important mechanism underlying the effects of SMF and paclitaxel on K562 cells.

Strieth et al. [98] analyzed the effects of SMF (≤587 mT) on tumor microcirculation. *In vivo* fluorescence microscopy was performed in A-Mel-3 tumors growing in dorsal skinfold chamber preparations of hamsters. Short time exposure to SMF (≥150 mT) resulted in a significant reduction of capillary red blood cells velocities (*v*RBC) and segmental blood flow in tumor microvessels. At the maximum strength of 587 mT, a reversible reduction of *v*RBC (40%) and of functional vessel densities (FVD) (15%) was observed. Prolongation of the exposure time (1 minute to 3 h) resulted in reductions. Microvessel diameters and leukocyte-endothelial cell interactions remained unaffected by SMF exposures. However, in contrast to tumor-free striated muscle controls, exposure at the maximum flux density of 587 mT induced a significant increase in platelet-endothelial cell adherence in a time-dependent manner that was reversible after reducing the strength of the SMF. The authors assumed that these reversible changes may have implications for functional measurements of tumor microcirculation by MRI and new therapeutic strategies using strong SMFs. The same research group further evaluated the effects of an SMF (586 mT, for 3 h) on tumor angiogenesis and growth [99]. Analysis of microcirculatory parameters revealed a significant reduction of FVD, vessel diameters, and RBC velocity in tumors after SMF exposure compared with the control tumors. These changes reflect retarded vessel maturation by antiangiogenesis. The increased edema after SMF-exposure indicated an increased tumor microvessel leakiness possibly enhancing drug uptake. The authors concluded that SMF therapy appears to be a promising new anticancer strategy, as an inhibitor of tumor growth and angiogenesis and as a potential sensitizer to chemotherapy.

Chen et al. [100] investigated whether 8.8 mT SMFs can enhance the killing potency of cisplatin (DDP) on human leukemic cells (K562). The results show that SMFs enhanced the anticancer effect of DDP on K562 cells. The mechanism correlated with the DNA damage model. This study also shows the potentiality of SMFs as an adjunctive treatment method for chemotherapy.

Hao et al. [83] investigated whether a moderate-intensity SMF can enhance the killing effect of ADM on K562 cells and explore the effects of SMF combined with ADM on K562 cells. The authors analyzed the metabolic activity of cells, cell cycle distribution, DNA damage, change in cell ultrastructure, and P-glycoprotein (P-gp) expression after K562 cells were exposed continuously to a uniform 8.8 mT SMF for 12 h, with or without ADM. Their results showed that the SMF combined with ADM (25 ng/mL) significantly inhibited the metabolic activity of K562 cells, while neither ADM nor the SMF alone affected the metabolic activity of these cells. Cell ultrastructure was altered in the SMF + ADM group. For example, cell membrane was depressed, some protuberances were observable, and vacuoles in the cytoplasm became larger. Cells were arrested at the G2/M phase and DNA damage increased after cells were treated with the SMF + ADM. ADM also induced the P-gp expression. In contrast, in the SMF group and SMF + ADM group, the P-gp expression was decreased compared with the ADM group. Taken together, these results showed that the 8.8 mT SMF enhanced the cytotoxicity potency of ADM on K562 cells, and the decrease in P-gp expression may be one reason underlying this effect.

Cells were treated with four anticancer drugs followed by treatment with a combination of drugs and SMF [101]. Individual cells were examined using atomic force microscopy (AFM). The drugs were taxol (alkaloid), doxorubicin (anthracycline), cisplatin (platinum compound), and cyclophosphamide (alkylating agent). Holes were observed in cells exposed to SMF but not in control groups. The number, size, and shape of the holes were dependent on the drug type, SMF parameters, and the duration of exposure. The results suggest that the application of a SMF could alter membrane permeability, increasing the flow of the anticancer drugs. This may be one of the reasons why SMF can strength then the effect of anticancer drugs. Observations were also made of the effect of using different anticancer drugs. For example, the effect of SMF combined with taxol or cyclophosphamide on the cells was additive while the effect of SMF combined with cisplatin or doxorubicin was synergistic. The target sites of cisplatin and doxorubicin are nucleic acids; continous research is required into this important area to ascertain the effect of SMF on nucleic acids. 

### 4.2. Strong and Ultrastrong Static Magnetic Fields and Cancer

Recently, some studies have suggested that SMFs have the potential as an adjunctive treatment method for chemotherapy, since SMFs influence cell growth, proliferation, and structure of cancer cells [98, 99, 102–107]. In particular, the killing effect of antineoplastic drugs on cancer cells is enhanced with a combined treatment of SMFs and chemotherapeutic drugs, indicating that SMFs act synergically with the pharmacological treatment [71, 96, 108]. For example, 64 h exposure to a 7 T uniform SMF produced a reduction in viable cell number in HTB 63 (melanoma), HTB 77 IP3 (ovarian carcinoma), and CCL 86 (lymphoma, Raji cells) cell lines [102].

Ghibelli et al. [109] examined whether exposure to the SMF of NMR (1 T) generated by an NMR apparatus can affect apoptosis induced on reporter tumor cells of hematopoietic origin. The impressive result was the strong increase (by 1.8–2.5-fold) of damage-induced apoptosis by NMR. This potentiation is due to cytosolic Ca^2+^ overload to NMR-promoted Ca^2+^ influx, since it is prevented by intracellular (BAPTA-AM) and extracellular (EGTA) Ca^2+^ chelation or by inhibition of plasma membrane L-type Ca^2+^ channels. A 3-day followup of treated cultures showed that NMR decreases long-term cell survival, thus increasing the efficiency of cytocidal treatments. Mononuclear white blood cells are not sensitized to apoptosis by NMR, showing that NMR may increase the differential cytotoxicity of antitumor drugs on tumor versus normal cells. The authors suggested that this strong, differential potentiating effect of NMR on tumor cell apoptosis may have important implications, as in fact a possible adjuvant for antitumor therapies.

## 5. Summary and Conclusions

In recent years, an abundance of research papers, review papers, and books has been published describing the possible physical and biological interactions of magnetic fields.

Considering these articles comprehensively, the conclusions are as follows: the primary cause of changes in cells after incubation in external SMF is disruption of free radical metabolism and elevation of their concentration. Such disruption causes oxidative stress and, as a result, damages ion channels, leading to changes in cell morphology and expression of different genes and proteins and also changes in apoptosis and proliferation. Moreover, based on available data, it was concluded that exposure to SMFs alone has no or extremely small effects on cell growth and genetic toxicity regardless of the magnetic density. However, in combination with other external factors such as ionizing radiation and some chemicals such as cadmium, there is evidence strongly suggesting that an SMF modifies their effects. Effects of SMFs on apoptosis are a potentially interesting phenomenon. However, these effects often depended on a cell type and were not found in various types of cells. Many researchers have observed the effects of SMFs on tumor cells, particularly the inhibiting effects. In order to reduce the toxicity and resistance of single anticancer drugs, a variety of unified treatments were required. The synergy of magnetic fields and anticancer drugs was one of the methods. It provides a new strategy for the effective treatment of cancer.

These studies provide valuable insight into the phenomenon of biomagnetism and open new avenues for the development of new medical applications. Further studies are necessary to explore the mechanisms of the SMF action in more detail.
